# Supplementary material for: Time spent by hospital personnel on drug changes: A time and motion study from an in-and outpatient hospital setting
Source: PLoS One. 2021 Feb 25;16(2):e0247499. doi: 10.1371/journal.pone.0247499 (PMC7906352; doi:10.1371/journal.pone.0247499)
Supplement: Attachment — Submitted filename: comments on PONE-D-20-21353.docx [file pone.0247499.s002.docx]

Data provided support the conclusions, however the data set is quite limited. The authors have taken a non-biased approach to measuring time impact, using eye-tracking technology rather than just direct observation – this is commendable. The appropriately cite the difficulty with other reports of time impact of drug changes using survey methods, which are affected by recall bias. Unlike most other publications that focus on pharmacy activities, this report focuses on nursing activity and the impact of drug changes. This situation may be more relevant to hospitals that put more drug control in the hands of pharmacy personnel and that use more automated systems, such as Automated Dispensing Units, barcode technology and even unit dose medications. Most North American institutions have medication orders written by generic name and drug selection is not required by nursing personnel. Thus drug changes (in brand provided) do not impact nursing time. The changes reported by the authors were not significant in all categories, with only semi-drug changes causing a significant increase in time spent by nursing – these changes were relatively short-lived, with time spent returning almost to baseline. IN the outpatient setting of rheumatology clinic, time was impacted more significantly, but for a brief interval only for drug changes and also for drug shortage situations, which may occur at any time of year, regardless of contract changes.

The authors suggest (lines 282-286) that system changes such as prescribing using generic names could prevent confusion during drug changes. Inventory management was briefly alluded to as the medication room continued to have stock of the previous brand, leading to incongruence between prescribed drug name and available drug name.

The authors suggest that administrators should be made aware of the need for additional time (resource) to adapt to drug changes, but they have not provided a financial cost for this additional resource by extrapolating their data to an entire hospital implementing numerous drug changes annually. Most administrators would require more evidence than this small sample size to justify changes in resource allocation.

It would have been interesting to have the researchers measure the time impact on nursing personnel, particularly in the outpatient rheumatology unit of switching brands of biologic medications from reference brand to biosimilar, as nursing education and support of patients is crucial for acceptance and adherence in that scenario.
