# Supplementary material for: Time spent by hospital personnel on drug changes: A time and motion study from an in-and outpatient hospital setting
Source: PLoS One. 2021 Feb 25;16(2):e0247499. doi: 10.1371/journal.pone.0247499 (PMC7906352; doi:10.1371/journal.pone.0247499)
Supplement: Attachment — Submitted filename: Response to Reviewers.docx [file pone.0247499.s003.docx]

Dear reviewers at PLOS ONE

Thank you for a thorough reading and constructive criticism of our manuscript entitled "Time spent by hospital personnel on drug changes: A time and motion study from an in-and outpatient hospital setting”. We are pleased with the opportunity to revise the original manuscript and we especially thank you for your relevant comments to the article. You have pointed out important aspects that needs clarifications, which we have tried our best to respond to. With these improvements, we hope to attract readers all over the health care sector, health authorities and the pharmaceutical industry.

It is with great pleasure that we send this rebuttal letter that responds to your suggested revision of the original manuscript for resubmission to PLOS ONE. We hope the revised article meets your expectations satisfactorily. Again, we thank you for your professional opinion, constructive feedback and input. Enjoy the revised article.

On behalf of all co-authors,

Yours sincerely,

Joo Hanne Poulsen

Journal Requirements: When submitting your revision, we need you to address these additional requirements.

1. Please ensure that your manuscript meets PLOS ONE's style requirements, including those for file naming. The PLOS ONE style templates can be found at

<https://journals.plos.org/plosone/s/file?id=wjVg/PLOSOne_formatting_sample_main_body.pdf> and

<https://journals.plos.org/plosone/s/file?id=ba62/PLOSOne_formatting_sample_title_authors_affiliations.pdf>

We have reformatted the manuscript according to the above style guidelines. More specifically, we have adjusted the front page “Amended title page, authors, affiliations” according to the formatting guideline. Specific supplements have been made in the author byline, particularly related to the symbol legend, just as the contributorship are stated more clearly.

Further, we have also changed the font type in the method section, just as changes in the figure and table captions appear in accordance to the manuscript body formatting guideline.

We hope that it now fits the style requirements, as described in the referred templates

2.  Thank you for stating the following in the Financial Disclosure section:

"JHP is a part of a co-financed PhD study funded by Amgros I/S, Copenhagen Academy for Medical Education and Simulation (CAMES), The Hospital Pharmacy in the Capital Region of Denmark and the University of Copenhagen. Peter Dieckmann holds a professorship at the University of Stavanger that is funded by an unconditional grant of the Laerdal Foundation (Stavanger, Norway) to the University of Stavanger. The funders had no role in study design, data collection and analysis, decision to publish, or preparation of the manuscript."

We note that you received funding from a commercial source: Amgros I/S.

Please provide an amended Competing Interests Statement that explicitly states this commercial funder, along with any other [relevant declarations](about:blank) relating to employment, consultancy, patents, products in development, marketed products, etc.

Within this Competing Interests Statement, please confirm that this does not alter your adherence to all PLOS ONE policies on sharing data and materials by including the following statement: "This does not alter our adherence to PLOS ONE policies on sharing data and materials.” (as detailed online in our guide for authors [http://journals.plos.org/plosone/s/competing-interests](about:blank)).  If there are restrictions on sharing of data and/or materials, please state these. Please note that we cannot proceed with consideration of your article until this information has been declared.

Please include your amended Competing Interests Statement within your cover letter. We will change the online submission form on your behalf.

Please know it is PLOS ONE policy for corresponding authors to declare, on behalf of all authors, all potential competing interests for the purposes of transparency. PLOS defines a competing interest as anything that interferes with, or could reasonably be perceived as interfering with, the full and objective presentation, peer review, editorial decision-making, or publication of research or non-research articles submitted to one of the journals. Competing interests can be financial or non-financial, professional, or personal. Competing interests can arise in relationship to an organization or another person. Please follow this link to our website for more details on competing interests: [http://journals.plos.org/plosone/s/competing-interests](about:blank)

Please see the ‘Cover letter_amended_jhp0000’ where a Competing Interests Statement is found at page 2.

[Note: HTML markup is below. Please do not edit.]

Reviewers' comments:

Reviewer's Responses to Questions

**Comments to the Author**

1. Is the manuscript technically sound, and do the data support the conclusions?

The manuscript must describe a technically sound piece of scientific research with data that supports the conclusions. Experiments must have been conducted rigorously, with appropriate controls, replication, and sample sizes. The conclusions must be drawn appropriately based on the data presented.

Reviewer #1: Partly

Reviewer #2: Partly

We hope that the revised manuscript appears technically sound, and that the data support the conclusions.

2. Has the statistical analysis been performed appropriately and rigorously?

Reviewer #1: No

Reviewer #2: Yes

Answer to reviewer #1: Please see the detailed comments under section 5, item 6.

3. Have the authors made all data underlying the findings in their manuscript fully available?

The [PLOS Data policy](https://eur02.safelinks.protection.outlook.com/?url=http%3A%2F%2Fwww.plosone.org%2Fstatic%2Fpolicies.action%23sharing&data=04%7C01%7Cjoo.hanne.poulsen%40sund.ku.dk%7Cd66426f69de84817acdd08d871fc1dbf%7Ca3927f91cda14696af898c9f1ceffa91%7C0%7C0%7C637384675684111077%7CUnknown%7CTWFpbGZsb3d8eyJWIjoiMC4wLjAwMDAiLCJQIjoiV2luMzIiLCJBTiI6Ik1haWwiLCJXVCI6Mn0%3D%7C1000&sdata=Ux1nN8vWy%2BSExTfsQZhTWULNiYmG1CLngD227h9EZZE%3D&reserved=0) requires authors to make all data underlying the findings described in their manuscript fully available without restriction, with rare exception (please refer to the Data Availability Statement in the manuscript PDF file). The data should be provided as part of the manuscript or its supporting information, or deposited to a public repository. For example, in addition to summary statistics, the data points behind means, medians and variance measures should be available. If there are restrictions on publicly sharing data—e.g. participant privacy or use of data from a third party—those must be specified.

Reviewer #1: Yes

Reviewer #2: Yes

4. Is the manuscript presented in an intelligible fashion and written in standard English?

PLOS ONE does not copyedit accepted manuscripts, so the language in submitted articles must be clear, correct, and unambiguous. Any typographical or grammatical errors should be corrected at revision, so please note any specific errors here.

Reviewer #1: Yes

Reviewer #2: Yes

5. Review Comments to the Author

Please use the space provided to explain your answers to the questions above. You may also include additional comments for the author, including concerns about dual publication, research ethics, or publication ethics. (Please upload your review as an attachment if it exceeds 20,000 characters)

Reviewer #1: The authors have performed an empirical time and motion study to identify the time spent by hospital personnel on drug changes. Data has been collected for inpatient (cardiology wing) and outpatient (rheumatology wing) of a Danish hospital. Have used certain statistical methods to compare the results. However, there are some major issues that have not been addressed in this paper.

1) The data has been collected by a single person in this study (JHP). This definitely leads to personal bias in all the data collection aspects. It would have been better if at least two members were involved in data collection stage.

We agree that this is a limitation in the study, which will be discussed more clearly in the revised manuscript under “strength and limitations”. In continuation of #reviewer comment 4), these limitations have been clarified and discussed in the revised manuscript.

We hope that it meets your comment satisfactorily.

2) The authors have mentioned that it would not be possible to measure the Hawthorne effect. But this is an experimental study which has been carried out using sophisticated techniques like Tobii eye tracker, Go pro camera etc. Unless the nurses and SHA are trained on these methods, the impact created by these equipment on the behaviour cannot be underestimated.

We agree that the Hawthorne effect should not be underestimated. The use of an eye tracker in this study has affected the nurses and SHA, but the magnitude of the effect is unfamiliar, as nurses and SHA could be either faster or slower in the presence of an observer, eye tracking and go-pro camera. However, we have stated this limitation more clearly in the revised manuscript.

*The extent of the Hawthorne effect is difficult to estimate in the study, but the observer is assumed to have affected the observed participants to some extent. However, the general impression is that this bias diminished over time, since the participants became more and more comfortable with being observed [32]. Additionally, the physical appearance of the go-pro camera and the eye-tracking glasses mounted to the participant’s head have indeed also affected the participants while being observed. However, just as the bias related to the observer’s presence, the general level of interference of the glasses is assumed to diminish over time as participants become more accustomed with the glasses. The Hawthorne effect in this matter is impossible to escape, unless the nurses and SHA became accustomed to working with the glasses.*

We hope that it meets your comment satisfactorily.

3) In the cardiology wing, data has been collected in March 2019 (baseline) and April-May 2019 (immediately after DCs are introduced). This is definitely not a good comparison period because the authors have taken a baseline period wherein the nurses and SHAs were very well versed with the existing drugs (March 2019), and the study period of April-May 2019 is a learning phase even for an experienced nurse/SHA. If the data was collected a few months after the drug change (DC), the actual impact of the time could have been assessed and concluded.

Thank you for your observation. We would like to emphasize that the purpose of the study was to explore the time spent by hospital personnel in a drug change situation. Thus, we were specifically interested in this phase immediately after a DC, which is the phase where the personnel gets familiar with the new drug. Furthermore, we were interested in exploring, whether the DS phase would persist long term, but this was only explored in the outpatient clinic. However, we acknowledge that the meaning of a “DC situation” may be unclear. Thus, in the introduction section (line 74) we attempt to clarify what a DC situation embodies (changes to drugs new/unfamiliar to the dispensing personnel).

*The focus of this study is the DC situation, meaning the actual changes to new drugs unfamiliar to the dispensing personnel.*

Further, in the discussion, an additional statement is applied to the fact that no follow-up from the cardiology ward was made.

*No follow-up at the cardiology ward took place and thus, claiming that the extra time spent on DCs are related to immediate expenses due to tender are not possible. However, the follow-up data from the rheumatology outpatient clinic indicates that the extra time observed in relation to DC diminished over time and returns to baseline. Thus, one could assume a similar trend at the cardiology ward as the nurses and SHA become more familiar with dispensing of the implemented drugs.*

We hope that our specifications meet your comment satisfactorily.

4) In pages 7 & 8 of the manuscript, the authors mention how the software used for collecting data of Tobii glass tracker works. "Potential irrelevant times have been discarded". The authors need to explain more on how they decided irrelevant time. Moreover observing times from video and using it for data collection purpose can include 'bias'. Need to be addressed appropriately.

We agree that there is a bias related to the use of observing time from video and using it as data. Further, having one researcher (JHP) performing the coding may have resulted in intra-rating bias. These limitations have been clarified and discussed in the revised manuscript.

*At the cardiology ward, the same person (JHP) performed all the observations and coding of data. This may have resulted in personal and intra-rating bias, which could potentially affect the reliability of the study [40]. Obviously, having two or more researcher performing the observations and coding independently, discuss their finding or calculating a coder reliability, would enhance the overall reliability and reduce potential personal bias in the study. One way to minimize the potential intra-rating bias is to reduce the risk of “coder-drift”, which refers to applying the coding differently due to boredom or fatigue when coding larger data set at once [40]. However, in this study the coding was performed immediately after each observation and thus potentially reduce intra-rating bias [40]. Further, figure 1 displays the coding frame for the observations hereby increasing the transparency of the coding of included and discarded time. Conversely, having the same person performing the data collection results in less observation bias and reliability issues compared to the outpatient clinic, where pharmacy internship students were involved too. Despite the guidance of students, there might be differences in the perception of the time registrations in the consultation elements. However, these differences are considered to have minor influence on the registered time differences and overall findings.*

Additionally, based on your relevant comment, figure 1 has been added to increase the transparency of the data analysis. Figure 1 provides an example of the coding of relevant and irrelevant time intervals related to medicine dispensing at the cardiology ward (line 160-165).

*The time division, also referred to as coding in this study, was performed immediately after each observation. An example of the coding of relevant and irrelevant time intervals related to medicine dispensing step 3-6 at the cardiology ward is provided in Fig 1.*

***Fig 1. Coding of relevant and irrelevant time intervals – an example.*** *An example of coding of relevant and irrelevant time intervals related to medicine dispensing at the cardiology ward.*

We hope that our specifications and supplement of the table to the manuscript meet your comment satisfactorily.

5) The authors should explain why they have used different data collection techniques for inpatient (eye tracker, video etc.) and outpatient (mobile stop watch) setting.

We agree with your comment. Hence, in the method section, the reasons for the different data collection techniques are highlighted further.

*As the dispensing process at the cardiology ward involves seconds, the use of a refined and detailed data collection technique is required. Thus, the eye tracking was used in this setting. Additionally, the dispensing process at the rheumatology outpatient setting contain other activities and patients, thus, the use of an overall time measurement, such as a stopwatch was more useful in this setting. The use of the details provided by the eye tracking were not relevant in outpatient setting, just as the physical presence of the glasses would induce a considerable disturbance in the patient consultation. Thus, different data collecting technique were used for in- and outpatient settings*.

We hope that our specifications meet your comment satisfactorily.

6) I did a quick data analysis for cardiology ward (baseline) and cardiology ward (comparison).The data points for comparison period is very less. More over, the authors have taken an "average of averages" for the time comparison. This is definitely not a correct practise. For instance, even in base line phase average time taken by nurse 5 is 30.7 sec and that of nurse 7 is 14 seconds. So nurses cannot be treated as "homogeneous units" in data analysis. Need more explanation on this.

Thank you for the comment. We acknowledge and agree that the nurses are not a homogeneous group, however; we are unsure what is meant by the “average of averages”. The supporting information contain raw data for baseline, comparison and follow-up (outpatient clinic) and from this raw data, averages are calculated. Thus, we do not calculate an average for one specific nurse and then calculate the averages for all nurses afterwards. Further, we are aware of the differences between the registered dispensing, thus, the standard deviation, as well as the minimum and maximum, are outlined in the manuscript. In these tables the variations between the different time points appear. Additionally, we do not focus on the time differences between the individual nurses at the cardiology ward. It is not of relevance to the study to adjust for the individual nurse before and after the DCs, as this would entail the dispensing of the exact same drug in both periods, which is not measured and thus not focused on in this study.

We are happy to present data in another way, however; a clarification of the comment in further details would be needed for us to comply to the comment satisfactorily.

Reviewer #2: Comments have been pasted from a Word document (attached) as info was lost due to the way the system saves info. Data provided support the conclusions, however the data set is quite limited.

We fully agree with the comment and we have stated that more clearly in the manuscript in terms of affecting the reliability of results.

*Even though the actual implementation of DCs were effective from April 1^st^, relatively few DCs were observed in the cardiology ward in the comparison period (April and May), which potentially affects the reliability of the results.*

We hope that our specifications meet your comment satisfactorily.

The authors have taken a non-biased approach to measuring time impact, using eye-tracking technology rather than just direct observation – this is commendable. The appropriately cite the difficulty with other reports of time impact of drug changes using survey methods, which are affected by recall bias. Unlike most other publications that focus on pharmacy activities, this report focuses on nursing activity and the impact of drug changes. This situation may be more relevant to hospitals that put more drug control in the hands of pharmacy personnel and that use more automated systems, such as Automated Dispensing Units, barcode technology and even unit dose medications. Most North American institutions have medication orders written by generic name and drug selection is not required by nursing personnel. Thus drug changes (in brand provided) do not impact nursing time. The changes reported by the authors were not significant in all categories, with only semi-drug changes causing a significant increase in time spent by nursing – these changes were relatively short-lived, with time spent returning almost to baseline. IN the outpatient setting of rheumatology clinic, time was impacted more significantly, but for a brief interval only for drug changes and also for drug shortage situations, which may occur at any time of year, regardless of contract changes.
The authors suggest (lines 282-286) that system changes such as prescribing using generic names could prevent confusion during drug changes. Inventory management was briefly alluded to as the medication room continued to have stock of the previous brand, leading to incongruence between prescribed drug name and available drug name.
The authors suggest that administrators should be made aware of the need for additional time (resource) to adapt to drug changes, but they have not provided a financial cost for this additional resource by extrapolating their data to an entire hospital implementing numerous drug changes annually. Most administrators would require more evidence than this small sample size to justify changes in resource allocation.

We fully agree with the comment. Thus, we have calculated and thus transitioned the reportable time spent on dispensing and extradition of medicine from in- and outpatient settings into economical expenses in USD. Details and additional sections are provided in order to calculate the economic impact. Thus, they are found in the method section for both sub-studies, a new economic impact section (in method and results), just as the discussion and conclusion of the economic impact is changed accordingly to the results.

We hope that this supplement meets your comment satisfactorily.

It would have been interesting to have the researchers measure the time impact on nursing personnel, particularly in the outpatient rheumatology unit of switching brands of biologic medications from reference brand to biosimilar, as nursing education and support of patients is crucial for acceptance and adherence in that scenario.

We agree with this perspective. It would have been highly interesting to explore DCs of biosimilar medicines from the perspectives of nursing and patient education, but the focus of this study was on general DCs due to tendering. A comment in the discussion section containing DC from originator to biosimilar are included. However, another study would be required in order to explore this perspective in details.

*Further, this study focusses on a generic DC due to tender, but one could image that dealing with DC from originator to biosimilar would require extra resources for nursing education and patient information, due to another complexity of the DC. However, another study would be required in order to explore this perspective in details.*

We hope that this supplement meets your comment satisfactorily.

6. PLOS authors have the option to publish the peer review history of their article ([what does this mean?](https://eur02.safelinks.protection.outlook.com/?url=https%3A%2F%2Fjournals.plos.org%2Fplosone%2Fs%2Feditorial-and-peer-review-process%23loc-peer-review-history&data=04%7C01%7Cjoo.hanne.poulsen%40sund.ku.dk%7Cd66426f69de84817acdd08d871fc1dbf%7Ca3927f91cda14696af898c9f1ceffa91%7C0%7C0%7C637384675684121075%7CUnknown%7CTWFpbGZsb3d8eyJWIjoiMC4wLjAwMDAiLCJQIjoiV2luMzIiLCJBTiI6Ik1haWwiLCJXVCI6Mn0%3D%7C1000&sdata=82rxmUGZxIH%2FqIwH8Kj56CPUmebqUskbpeDI8pk7mWw%3D&reserved=0)). If published, this will include your full peer review and any attached files.

If you choose “no”, your identity will remain anonymous but your review may still be made public.

**Do you want your identity to be public for this peer review?** For information about this choice, including consent withdrawal, please see our [Privacy Policy](https://eur02.safelinks.protection.outlook.com/?url=https%3A%2F%2Fwww.plos.org%2Fprivacy-policy&data=04%7C01%7Cjoo.hanne.poulsen%40sund.ku.dk%7Cd66426f69de84817acdd08d871fc1dbf%7Ca3927f91cda14696af898c9f1ceffa91%7C0%7C0%7C637384675684121075%7CUnknown%7CTWFpbGZsb3d8eyJWIjoiMC4wLjAwMDAiLCJQIjoiV2luMzIiLCJBTiI6Ik1haWwiLCJXVCI6Mn0%3D%7C1000&sdata=t%2FsN4lrOK5k5lHWObtw7gd9N7BxX8MYq1Spfq6N0pGU%3D&reserved=0).

Reviewer #1: No

Reviewer #2: No

[NOTE: If reviewer comments were submitted as an attachment file, they will be attached to this email and accessible via the submission site. Please log into your account, locate the manuscript record, and check for the action link "View Attachments". If this link does not appear, there are no attachment files.]

While revising your submission, please upload your figure files to the Preflight Analysis and Conversion Engine (PACE) digital diagnostic tool, [https://pacev2.apexcovantage.com/](https://eur02.safelinks.protection.outlook.com/?url=https%3A%2F%2Fpacev2.apexcovantage.com%2F&data=04%7C01%7Cjoo.hanne.poulsen%40sund.ku.dk%7Cd66426f69de84817acdd08d871fc1dbf%7Ca3927f91cda14696af898c9f1ceffa91%7C0%7C0%7C637384675684131070%7CUnknown%7CTWFpbGZsb3d8eyJWIjoiMC4wLjAwMDAiLCJQIjoiV2luMzIiLCJBTiI6Ik1haWwiLCJXVCI6Mn0%3D%7C1000&sdata=67AVX3SL3vOCVai5PxqwA%2FRQfQ6dWp0JJwCCTjmjQK0%3D&reserved=0). PACE helps ensure that figures meet PLOS requirements. To use PACE, you must first register as a user. Registration is free. Then, login and navigate to the UPLOAD tab, where you will find detailed instructions on how to use the tool. If you encounter any issues or have any questions when using PACE, please email PLOS at [figures@plos.org](mailto:figures@plos.org). Please note that Supporting Information files do not need this step.
