# Supplementary material for: Time spent by hospital personnel on drug changes: A time and motion study from an in-and outpatient hospital setting
Source: PLoS One. 2021 Feb 25;16(2):e0247499. doi: 10.1371/journal.pone.0247499 (PMC7906352; doi:10.1371/journal.pone.0247499)
Supplement: Attachment — Submitted filename: Response to Reviewer #1 at Plos One.docx [file pone.0247499.s004.docx]

Dear reviewer #1 at PLOS ONE

Once again, thank you for a thorough reading and constructive criticism of our manuscript entitled "Time spent by hospital personnel on drug changes: A time and motion study from an in-and outpatient hospital setting”. We thank you for your comments to the article, and we have tried our best to respond to the aspects that needs further clarifications.

It is with great pleasure that we send this rebuttal letter that responds to your suggested revision of the original manuscript for resubmission to PLOS ONE. We hope the revised article meets your expectations satisfactorily.

On behalf of all co-authors,

Yours sincerely,

Joo Hanne Poulsen

Additional Editor Comments (if provided):

Reviewer 2 is satisfied with the changes made; I hope you can respond to Reviewer 1's remaining questions.


[Note: HTML markup is below. Please do not edit.]

Reviewers' comments:

Reviewer's Responses to Questions

**Comments to the Author**

1. If the authors have adequately addressed your comments raised in a previous round of review and you feel that this manuscript is now acceptable for publication, you may indicate that here to bypass the “Comments to the Author” section, enter your conflict of interest statement in the “Confidential to Editor” section, and submit your "Accept" recommendation.

Reviewer #1: (No Response)

Reviewer #2: All comments have been addressed

We hope that the revised manuscript is now acceptable for publication.

2. Is the manuscript technically sound, and do the data support the conclusions?

The manuscript must describe a technically sound piece of scientific research with data that supports the conclusions. Experiments must have been conducted rigorously, with appropriate controls, replication, and sample sizes. The conclusions must be drawn appropriately based on the data presented.

Reviewer #1: Partly

Reviewer #2: Yes

We hope that the revised manuscript appears technically sound, and that the data support the conclusions

3. Has the statistical analysis been performed appropriately and rigorously?

Reviewer #1: I Don't Know

Reviewer #2: Yes

We believe that the statistical analysis that have been performed are appropriate and relevant and we hope that the revised manuscript is found acceptable for publication.

4. Have the authors made all data underlying the findings in their manuscript fully available?

The [PLOS Data policy](https://eur02.safelinks.protection.outlook.com/?url=http%3A%2F%2Fwww.plosone.org%2Fstatic%2Fpolicies.action%23sharing&data=04%7C01%7Cjoo.hanne.poulsen%40sund.ku.dk%7Cd66426f69de84817acdd08d871fc1dbf%7Ca3927f91cda14696af898c9f1ceffa91%7C0%7C0%7C637384675684111077%7CUnknown%7CTWFpbGZsb3d8eyJWIjoiMC4wLjAwMDAiLCJQIjoiV2luMzIiLCJBTiI6Ik1haWwiLCJXVCI6Mn0%3D%7C1000&sdata=Ux1nN8vWy%2BSExTfsQZhTWULNiYmG1CLngD227h9EZZE%3D&reserved=0) requires authors to make all data underlying the findings described in their manuscript fully available without restriction, with rare exception (please refer to the Data Availability Statement in the manuscript PDF file). The data should be provided as part of the manuscript or its supporting information, or deposited to a public repository. For example, in addition to summary statistics, the data points behind means, medians and variance measures should be available. If there are restrictions on publicly sharing data—e.g. participant privacy or use of data from a third party—those must be specified.

Reviewer #1: Yes

Reviewer #2: Yes

5. Is the manuscript presented in an intelligible fashion and written in standard English?

PLOS ONE does not copyedit accepted manuscripts, so the language in submitted articles must be clear, correct, and unambiguous. Any typographical or grammatical errors should be corrected at revision, so please note any specific errors here.

Reviewer #1: Yes

Reviewer #2: Yes

6. Review Comments to the Author

Please use the space provided to explain your answers to the questions above. You may also include additional comments for the author, including concerns about dual publication, research ethics, or publication ethics. (Please upload your review as an attachment if it exceeds 20,000 characters)

Reviewer #1: I have gone through the comments that you have provided. There are a few unanswered questions.

1) Can you please clarify the objective of the study? If the focus is only on seeing the time change during DC, it is very important to explain the duration of this effect? For example, say if a new drug change has happened on April 1st, till what time do you observe this increased drug dispensing time? Is it till May 1st or June 1st etc.

Thank you for your comment. We would like to emphasize that the aim of the study is to explore time spend by hospital personnel in a drug change situation when dispensing medicine to in- and outpatients in a hospital setting in the Capital Region of Denmark. Thus, the focus of this study is on the specific DC situation, meaning the time where the drugs are actually changing to new drugs unfamiliar to the dispensing personnel. We acknowledge that it would be an interesting aspect to know the duration of the effect, but our study was not designed to answer this question.

In this study, we have thus not explored the length or duration of the period of ‘semi-drug change’ and DC. Therefore, we are unable to answer how long the duration of the effect on dispensing time that we have observed is. In the study, we assume a 4 week period of ‘semi-drug change’ and DC when calculating the economic impact, but the actual duration of the periods are unknown with our dataset. Thus, based on your comment, we will emphasize this further in the discussion of the economic impact (line 370-380).

*At the cardiology ward, a total of 27 nurses and SHA’s cover the dispensing process in both day and evening shifts. Estimating that ‘semi-drug change’ and DC affect dispensing time in a period of 4 weeks leads to a calculated extra time of 2 hours and 22 minutes or 196 USD spent on the assumed ‘semi-drug change’ and DC at this particular cardiology ward. This extra time and expenses does not immediately seem to have a clinical and economic impact, especially not on a daily basis with extra time corresponding to 303 seconds and 7 USD. However, the size of the cardiology ward is assumed to cover a standard medical ward in the Capital Region of Denmark. Thus, multiplication of the expenses with the total number of medical wards will show a considerably amount of additional expenses spent locally, regionally or even nationally. It should be emphasized that the estimated 4 weeks period with ‘semi-drug change’ and DC are based on assumptions, since the actual duration of the periods are unknown in this study.*

We hope that our specifications meet your comment satisfactorily.

2) You mention that ". The extent of the Hawthorne effect is difficult to estimate in the study, but the observer is assumed to have affected the observed participants to some extent. However, , but the general impression is that this bias diminished over time, since the participants became more and more comfortable with being observed"?

What is the meaning diminished over time? This is again connected to previous question. If this claim has to be made, then you need to have the following data. Before drug change, identify the base line time for dispensing with and without observer. Quantify the difference between the two. Once you notice that there is no statistical difference in the base line time of dispensing for baseline (with and without observer), you need to look for effect in situations of drug change. I comment this based on your results in Table 3. For baseline, you have 20.5 +/- 10.5 as the time. However after DC it is 28.4 +/- 16.3. Higher standard deviation can be due to presence of observer or due to the change in drugs. This is definitely not a good thing. Again, you do not mention how many days did this increased time (28.4 sec) lasted after DC.

Thank you for your comment. We have chosen to delete and re-write the sentence to meet your comment (line 418-428). We do not measure the Hawthorne effect, and therefore we do not know whether or not it diminishes over time, thus we simply wishes to acknowledge that it is theoretically present. However, as the same participants and observer is present in baseline and comparison, we assume that the Hawthorne effect is equally widespread in both data collection periods. In addition to this assumption, we have not changed any procedures or done anything differently in the data collection periods that can either minimize, maximize or affect the Hawthorne effect from one period to another.

*The extent of the Hawthorne effect is difficult to estimate in the study, but the observer is assumed to have affected the observed participants to a minor extent. Additionally, the physical appearance of the go-pro camera and the eye-tracking glasses mounted to the participant’s head have indeed also affected the participants while being observed. The Hawthorne effect in this matter is impossible to avoid, unless the nurses and SHA became accustomed to working with the glasses. Further, as the same participants and observer is present in baseline and comparison, the Hawthorne effect is assumed equally widespread in both data collection periods, although the effect has not been measured in this study.*

In terms of the standard deviations (SD) in baseline versus comparison, we acknowledge that there is a difference in SD’s. Based on the already assumed Hawthorne effect in both data collection periods, we do not assume that the Hawthorne effect has had a significant impact on SD. With regard to DCs, we agree that the effect on both mean and SD can be due to the change in drugs or ‘semi-drug change’. Another relevant factor that may impact the SD is the sample size. In baseline, the sample size is N = 161, whereas it is N = 35 (DCs and ‘semi-drug changes’) in comparison, and thus the calculation of SD in comparison is based on a relatively small sample size, which implies a larger SD. We have not included this in the discussion as it is a statistical technicality and the study was not designed to collect generalizable data and thus no power calculation were performed before data collection.

With regard to the duration of the effect on dispensing time we hope that our provided specifications meet the comment by Reviewer #1 satisfactorily.

3) Figure 1 is unclear. Please provide a better quality picture and an example of timelines for a particular scenario.

Thank you for your observation. Smaller corrections to figure 1 have been made in order to demonstrate more clearly how the coding of relevant and irrelevant time intervals related to dispensing at the cardiology ward have been divided and the time included or discarded in the study. An example of a time line for a particular scenario covering the dispensing process step 3-6 for Hexalactone has been provided (line 157-162).

*An example of the coding of relevant and irrelevant time intervals related to medicine dispensing in the cardiology ward for a particular scenario covering the dispensing process step 3-6 for Hexalactone has been provided in Fig 1.*

***Fig 1. Coding of relevant and irrelevant time intervals – an example.*** *An example of coding of relevant and irrelevant time intervals related to medicine dispensing at the cardiology ward for a particular scenario covering the dispensing process step 3-6 for Hexalactone.*

We hope that the quality of figure 1 is more clear now and that the amendments meet the reviewers comment satisfactorily.

4) To add further, since I noticed that the standard deviation is higher in DC, I wonder if it would be due to the effect of uncertainty in the measurements in itself. Can you please provide how much is the uncertainty of the measurement system?

Thank you for the comment. We are not entirely sure what is meant with the “uncertainty of the measurement system”. Throughout the data collection, the same measurement methods in the in- and outpatient setting respectively have been used. Thus, we assume that the measurement methods hold the same uncertainty in both baseline and comparison.

Reviewer #2: The authors have addressed my comments very well. The paper reads well and the authors have made adjustments based on other reviewer comments also. The strengths and limitations section as well as economic impact have been enhanced.

Thank you very much for review and constructive feedback.
